# Supplementary material for: Capsule networks as recurrent models of grouping and segmentation
Source: PLoS Comput Biol. 2020 Jul 21;16(7):e1008017. doi: 10.1371/journal.pcbi.1008017 (PMC7394447; doi:10.1371/journal.pcbi.1008017)
Supplement: S1 Appendix — (PDF) [file pcbi.1008017.s001.pdf]

S1 Appendix: Results are robust against hyperparameter changes

To show that the reproduction of (un)crowding is a general trait of CapsNets, we trained more networks with different hyperparameter sets both for experiment 1 (Fig A) as well as experiment 2 (Fig B). The following table summarizes all CapsNet hyperparameters used for the results in the main text, as well as in the following two figures.

Fig A shows that our results in experiment 1 are robust against changes in all these network hyperparameters. This diversity highlights that our results are not the outcome of one particular set of overfitted hyperparameters. Rather, it suggests that the grouping and segmentation mechanisms in CapsNets can naturally explain the occurrence of crowding and uncrowding in our datasets. The robustness of these results stresses the importance of the underlying network architecture for (un)crowding. Few networks were excluded because vernier discrimination performance in the vernier-alone condition was floored (<=55%).

Fig B shows that our results in experiment 2 are robust against hyperparameter changes as well. We also found that small changes in the stimuli, such as changing the cuboids from opaque to transparent, does not affect the results either (compare Fig A). In both of the given examples, the improvement in vernier discrimination performance with an increasing number of recurrent routing iterations was greater in the cuboids condition than in the lines condition (a: $p=0.013$, b: $p=0.007$, 2-tailed two-sample t-tests). This stresses the importance of the underlying network architecture for reproducing the temporal dynamics of (un)crowding. Some networks were excluded because vernier discrimination performance for the line and cuboid flankers was at ceiling (>=95%) or floor (<=55%).

	Exp 1 Main text	Exp 2 Main text	Exp 1 S1 Fig 1a	Exp 1 S1 Fig 1b	Exp 1 S1 Fig 1c	Exp 1 S1 Fig 1d	Exp 2 S1 Fig 2a	Exp 2 S1 Fig 2b
Input image size	20 x 72	16 x 48	20 x 72	16 x 72	20 x 72	30 x 72	16 x 48	16 x 48
Shape size	14x14	14x11x6	14x14	14x14	14x14	14x14	14x11x6	14x11x6
Number of shape types	7	4	7	4	4	4	4	4
Shape repetitions	[1, 3, 5]	2	[1, 3, 5]	[1, 3, 5]	[1, 3, 5]	[1, 3, 5]	2	2
Number convolutional layers	3	3	3	3	3	3	3	3
Kernel sizes	5, 5, 6	4, 4, 5	5, 5, 6	5, 5, 6	5, 5, 6	5, 5, 4	4, 4, 5	4, 4, 5
Strides	1, 1, 2	1, 1, 2	1, 1, 2	1, 1, 2	1, 1, 2	1, 2, 2	1, 1, 2	1, 1, 2
Dropout used?			x	x	x	x		
Primary capsule types	7	4	7	4	4	8	4	4
Total number primary capsules	840	228	840	240	480	480	228	228
Primary capsule dimensions	2	1	2	1	1	1	1	1
Secondary capsule types	7	4	7	4	4	4	4	4
Secondary capsule dimensions	8	3	10	4	4	12	5	7
Routing iterations	3	8	3	4	4	4	8	8
Networks trained	10	50	10	20	20	20	50	50
Networks after cleaning	10	43	10	19	19	20	42	48
Gaussian training noise	[0.02, 0.04]	[0.00, 0.02]	[0.02, 0.04]	[0.0, 0.02]	[0.0, 0.0]	[0.0, 0.0]	[0.0, 0.02]	[0.02, 0.04]
Contrast adjustment delta	[0.6, 1.2]	[0.9, 1.1]	[0.6, 1.2]	[0.6, 1.2]	[0.6, 1.2]	[0.8, 1.1]	[0.9, 1.1]	[0.9, 1.1]
Gaussian test noise	[0.04, 0.06]	[0.10, 0.12]	[0.04, 0.06]	[0.1, 0.12]	[0.0, 0.02]	[0.01, 0.01]	[0.2, 0.22]	[0.14, 0.16]
Batch size	48	48	48	48	48	48	48	48
Learning rate	0.0004	0.0004	0.0004	0.0004	0.0004	0.0004	0.0004	0.0004
Learning rate decay steps	250	500	250	300	200	250	300	350
Training steps	2500	4500	5000	3000	2000	3000	3000	3500
Vernier offset decoder used?	x	x	x	x	x	x	x	x
Reconstruction decoder used?	x	x	x				x	x
Shape repetition decoder used?	x		x	x	x	x		
Location decoder used?	x		x					

Table A: Network hyperparameters. The rows show all hyperparameters and the columns show their selected values for the different networks. Parameters for the main text results are highlighted. The number of convolutional layers includes the primary capsule layer. The total number of primary capsules is equal to the output size of the last convolutional layer multiplied with the number of primary capsule types

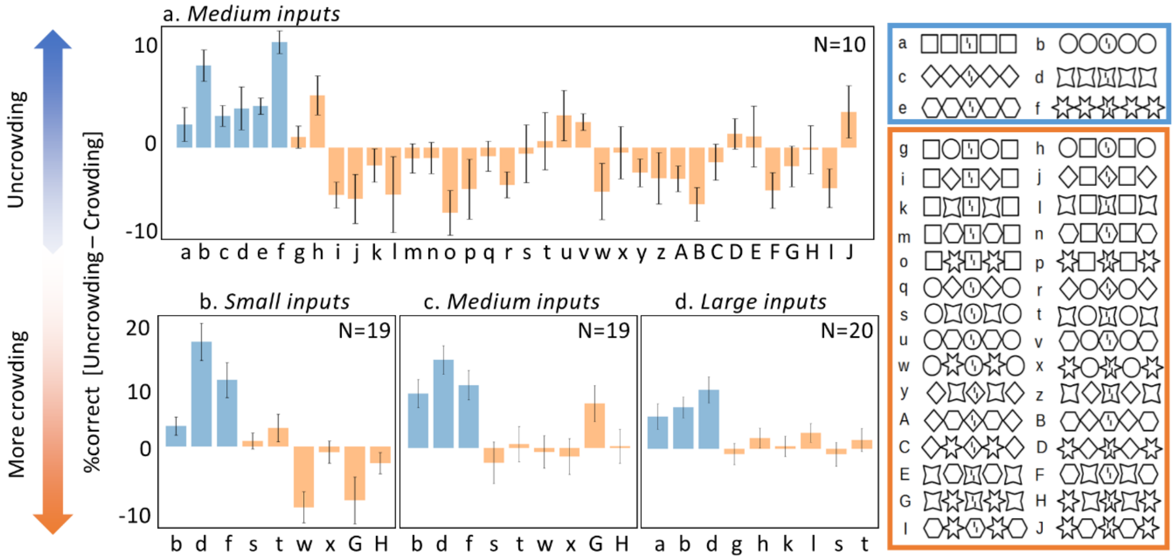

Fig A: Results are robust against hyperparameter changes in experiment 1. The x-axes show the configurations that were used and the y-axes show the vernier discrimination performance as %correct for the whole configuration minus %correct for the central flanker alone. Error bars indicate the standard error between trained networks. Crowding and uncrowding occur qualitatively similar in all networks with different hyperparameters using (b) small input images of size 16x72 pixels, (a, c) medium input images of size 20x72 pixels, and (d) larger input images of size 30x72 pixels.

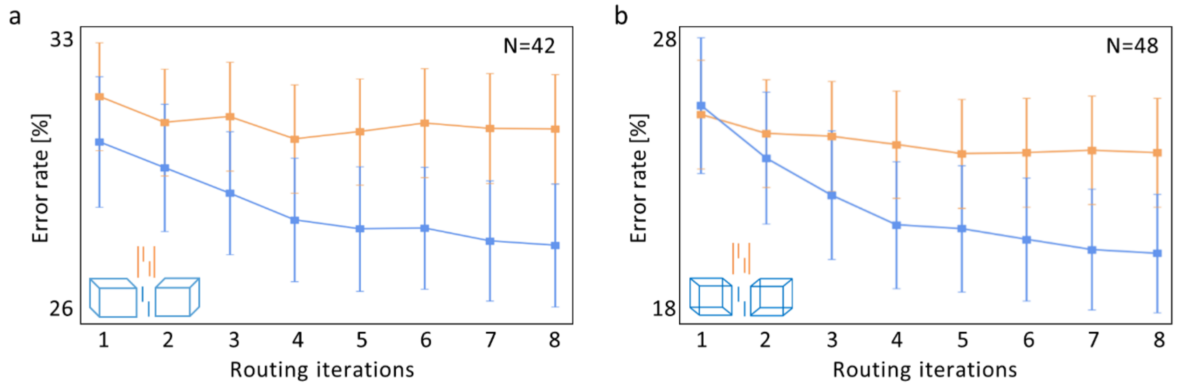

Fig B: Results are robust against hyperparameter changes in experiment 2. The x-axis shows the number of recurrent routing iterations and the y-axis shows the error rates for the vernier discrimination performance (i.e., lower values indicate better performance). Error bars indicate the standard error between trained networks. As for the main results, performance increases significantly more with routing iterations in the cuboids condition (blue) as compared to the lines condition (orange), reproducing human behavior.
