# Supplementary material for: Capsule networks as recurrent models of grouping and segmentation
Source: PLoS Comput Biol. 2020 Jul 21;16(7):e1008017. doi: 10.1371/journal.pcbi.1008017 (PMC7394447; doi:10.1371/journal.pcbi.1008017)
Supplement: S2 Appendix — (PDF) [file pcbi.1008017.s002.pdf]

S2 Appendix: Loss evolution during training

To exemplify the evolution of the loss, Fig A shows the progress of each loss term during training for each of the N=10 networks of the main results of experiment 1. The loss terms were scaled to ensure that none of them was dominant during training. All losses were minimized simultaneously.

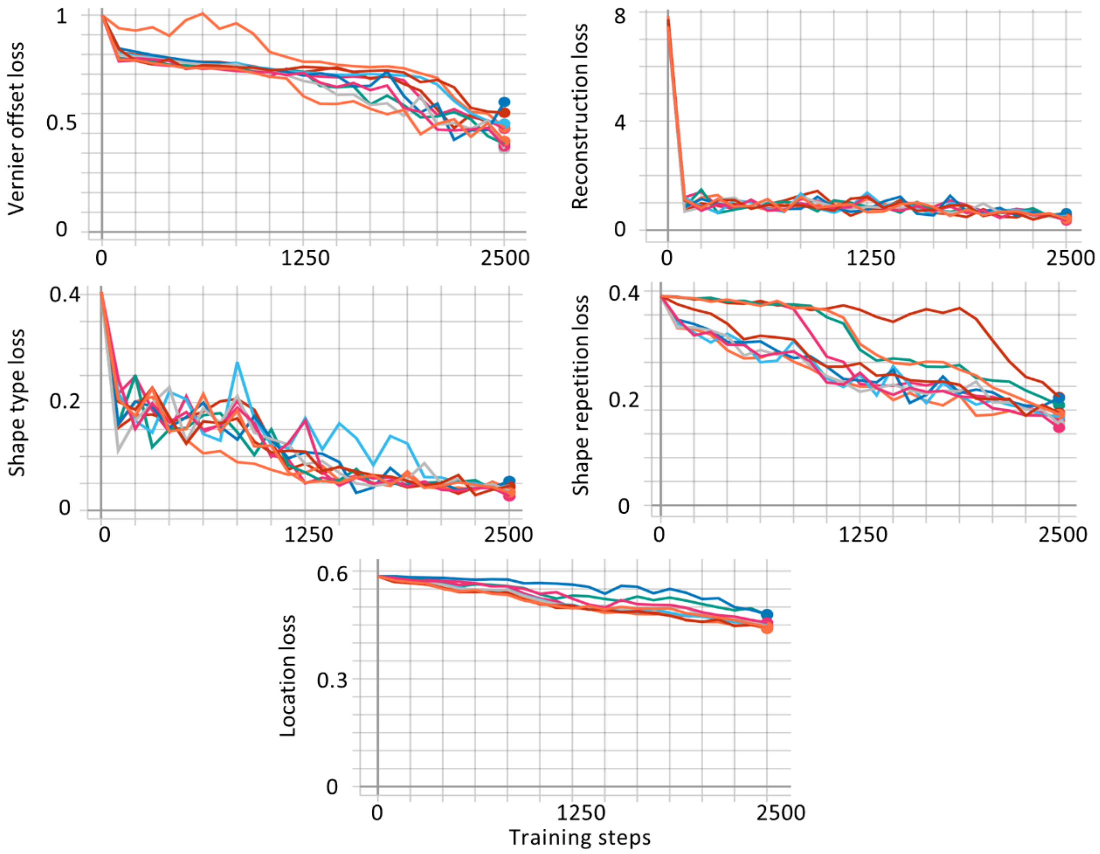

Fig A: Loss evolution during training. The x-axis shows the number of training steps and the y-axes shows the absolute loss values for each loss term. Each color represents one of the N=10 networks that was trained for the main results of experiment 1. All losses decreased during training.
