# Supplementary material for: Capsule networks as recurrent models of grouping and segmentation
Source: PLoS Comput Biol. 2020 Jul 21;16(7):e1008017. doi: 10.1371/journal.pcbi.1008017 (PMC7394447; doi:10.1371/journal.pcbi.1008017)
Supplement: S3 Appendix — (PDF) [file pcbi.1008017.s003.pdf]

S3 Appendix: Performance deteriorates due to crowding

To show that crowding occurred in our networks due to the proximity of the vernier target and the flankers, we ran a control experiment in which the vernier was presented outside the flanking shapes. Vernier discrimination accuracy did not drop in this case. This suggests that the performance drop can be ascribed to crowding and not some other peculiarity in the networks.

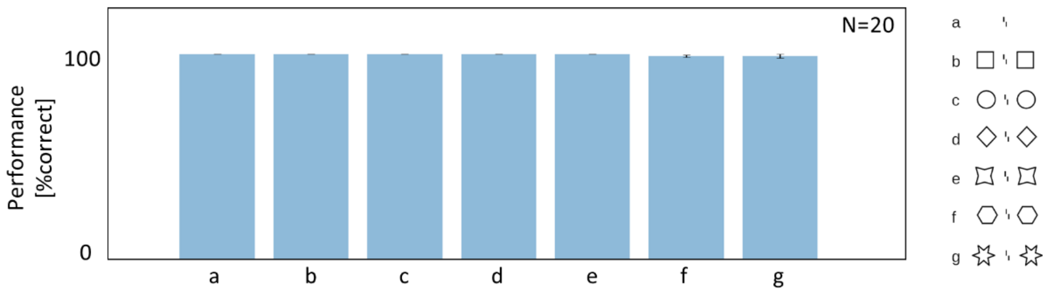

Fig A: Performance deteriorates due to crowding. The x-axis shows the configurations that were used and the y-axis shows the vernier discrimination performance. Error bars indicate the standard error between trained networks. Vernier discrimination accuracy does not decrease when the vernier is presented outside the flanking shapes (configurations b-g) compared to the vernier alone condition (configuration a).
