# Supplementary material for: Capsule networks as recurrent models of grouping and segmentation
Source: PLoS Comput Biol. 2020 Jul 21;16(7):e1008017. doi: 10.1371/journal.pcbi.1008017 (PMC7394447; doi:10.1371/journal.pcbi.1008017)
Supplement: Attachment — Submitted filename: Doerig_Schmittwilken_etal_Response_to_reviewers.docx [file pcbi.1008017.s004.docx]

We thank the editor and both reviewers for their feedback, which helped us improve and clarify the manuscript. We addressed all the points raised by the reviewers, and carried out some additional small improvements to the figure layouts and the language. These improvements do not alter the points we make, but they improve clarity. We also reran our simulation without using dropout, as proposed by reviewer 1. We have cleaned and commented the code to reproduce all our results and uploaded it here: <https://github.com/adriendoerig/Capsule-networks-as-recurrent-models-of-grouping-and-segmentation>. We also include the human behavioral data for experiment 2 in the same repository.

Reviewer 2 expressed some concerns that our results may potentially not be sufficiently novel with respect to a previous publication. However, there is very little overlap between the two except that psychophysical data come from similar experiments. Whereas the previous contribution was mainly about which models can explain crowding, this manuscript is about global processing and how shortcomings of standard ffCNNs in this domain can be overcome. Since global processing is one of the major hurdles facing current ffCNNs, this manuscript makes an important contribution to the fields of vision science, neuroscience and deep learning.

Neuroscience is being deeply impacted by artificial neural networks. However, comparisons between these networks and human processing are usually quite unspecific. Typically, large ffCNN models are compared by fitting large datasets. To our knowledge, there is little research on specific kinds of computations that ffCNNs can or cannot do, and, importantly, why. Here, we show that recurrent computations specifically implementing grouping and segmentation are needed to explain data from complex contextual human vision. Furthermore, we show that the computational dynamics are well modelled too. For this, we created a psychophysical experiment, which we and reviewer 1 think is interesting in itself because it goes beyond the usual static image recognition measures by taking time-extended computations into account. Strikingly, we found that increasing the time allowed for recurrent computations leads to a better segmentation of the flankers from the target in both humans and CapsNets (but not in ffCNNs and recurrent CNNs). This occurs through a dynamic process during which groups of neurons determine how to segment a stimulus in order to maximize agreement between capsules. Our results pave a new way for challenging neural networks by explicitly looking into the dynamics of processing. We think that paradigms such as ours may become important testbeds for neural network models in general.

As reviewer 1 noted, there are very few attempts to explain the dynamics of human neural computations using deep learning (with the notable exception of Kietzmann et al. 2019). This may be because most researchers use ffCNNs, which are ill-suited to address such dynamic computations. Thus, recurrent networks are needed. However, as we mention in our discussion, recurrent networks are still rarely used in neuroscience, and most recurrent networks that are used are simple recurrent extensions of ffCNNS without a specific computational purpose. In fact, this is the first time that CapsNets, which use specific interpretable recurrent processing, are used in a neuroscience context (to our knowledge). We had to develop a large code database to implement CapsNets and test them on psychophysical stimuli, decode vernier offsets, apply the right loss functions, investigate dynamic changes in capsule representations over time and to compare CapsNets with other network architectures.

Importantly, rather than a model geared at explaining one specific psychophysical result, CapsNets offer a general framework to understand human global processing in a wide variety of tasks and modalities (contrary to the LAMINART and its hand-tuned neural connections directly aimed at solving visual uncrowding). Indeed, CapsNets can be applied to any task in principle and are general enough to be considered as an alternative framework to the classic conception of the visual system embodied by ffCNNs. Our strong results show that such a framework is a qualitatively better model of human global processing. Future work is needed to tell to what extent CapsNets match human processing in different tasks, opening up exciting new research directions.

In summary, our main aim was not only to show that CapsNets can reproduce the (un)crowding results, but to understand how they do it and which kind of recurrent processing is needed in order to do so. This offers a fresh way of conceptualizing global visual processing and has important implications far beyond crowding, for neuroscience and computational science. We think that, when complex deep networks such as CapsNets scale, important new avenues of research will open up, and this is an early example of this promising approach. We added a new paragraph to the discussion explicating some of these points.

In the following, we respond to each comment of the reviewers in detail. The reviewers’ comments are in **bold** and our response in normal font.

**REVIEWER 1:**

**I have enjoyed reading this paper. The authors are probing the usability of capsule networks on a task they are supposed to (and actually do) solve, spatial configurations, with, as an emergent feature, behavior (mostly) matching (published) human perception on the occurrence of crowding and uncrowding.**

**To link processing in capsule networks to the type of computations involved in recurrent processing the authors conduct a second series of experiments. When humans are presented with a masking stimulus that is presumed to require recurrent processing to resolve, uncrowding emerges for a longer stimulus duration. This also occurs in capsule networks with more routing iterations.**

**This is a timely paper extending the range of AI networks being used to probe cognition. Also, experiment 2 has the potential of becoming an instant citation classic (I will certainly also include it in lectures to illustrate the types of experiments you can do with DNNs).**

Thank you very much for these comments.

**Major points**

**I a missing a lot of details on the network and have encountered some inconsistencies.**

Thank you for pointing out this lack of details. We added a table to the supplementary material, summarizing all parameters of our networks.

**• Can you indicate, also in the figure, how the loss is propagated from the loss to the output? Specifically, how are the different sources of loss inserted into the output layer.**

We updated Figure 5 (now called Figure 6 due to some minor rearranging in the rest of the text) to include all decoders, and show the equations for each decoder loss in the main text.

**• Caps of fig 5a and L373 refer to 3 convolutional layers on the text, the graphic, and main text to two layers. I suspect the latter. What are the dimensions of the input images? What are the specs of the convolutional layers? M refers to number of shape types. What is N exactly? How is it determined?**

There are two convolutional layers, followed by the primary capsules and finally the secondary (output) capsules. The primary capsules are implemented, as in standard capsule networks, by a convolutional layer that is reshaped to yield M capsule types, of N dimensions. Hence, in total, there are 2 convolutional layer, plus a 3^rd^ convolutional layer that is reshaped to yield the primary capsules. We clarified this in the methods and in the caption of figure 5 (now called Figure 6 due to some minor rearranging in the rest of the text).

All the parameters of our networks are now specified in the parameters table.

**• What are the other parameters of the conv layers?**

All the parameters of our networks are now specified in the parameters table.

**• If there are 3 conv layers this would be somewhat remarkable given the input images. Could you provide more details on why 3 are needed?**

3 convolutional layers (i.e., 2 normal conv. layers + 1 for primary capsules) was a convenient way of bringing the size of the primary capsule layer to a manageable size without using huge strides or pooling which would lose information. However, in our experience, details such as 2 vs. 3 convolutional layers does not heavily change the results.

**• Fig 5a refers to secondary capsules, I suspect these are the output capsules (but not the output layer).**

Thanks for pointing out this inconsistency. We now refer to secondary capsules throughout the manuscript.

**• Please have fig 5a include the entire network including, including the output layer (and how it relates to the different loss functions).**

We updated Figure 5 (now called Figure 6 due to some minor rearranging in the rest of the text) to include the entire network, including all output decoders, and the loss equations associated with each decoder are given in the main text. This was the best way we found to include all information in the clearest way.

**• L373 states there are dropout layers between the thee convolutional layers. I suspect this is not possible.**

Thank you for this comment. We used dropout because Kumar & Jadon (2019) mentioned that it helped learning in capsule networks. However, we agree that using dropout between convolutional layers may not be optimal. Therefore, we ran simulations without the dropout and found that, in fact, performance is largely more stable than with our previous networks that included dropout. Therefore, we now show the simulations without dropout in the main text (and moved some results with dropout to the supplementary material). Thank you again for this remark.

**• What is the development of loss during training? How is the loss of the other networks?**

All loss curves during training for each network of experiment 1 are now included in the supplementary material.

**The results from the psychophysical experiment appear to be brilliant, but only two lines. It would be even more convincing / impactful with an additional point. What would a Necker cube do? What would a square do?**

Thank you for these suggestions, which we plan to explore in future work.

**• Could you speculate further on how you see the relationship between capsule networks, recurrent processing and the brain. Do the authors think that capsule networks are a way (of many) in which grouping can be solved? L351-L366 shows signs of redaction after earlier reviewer comments but ends up at: things could be something. I realize different reviewers have different comments but I would invite the authors to take more of a position.**

Our main points are that grouping and segmentation are crucial for global shape processing, and that deep learning is flexible enough to investigate processes, such as grouping and segmentation, that go far beyond what is implemented in current ffCNNs. We do not claim that Capsule networks are the only way to solve grouping and segmentation, but they are an interesting avenue of research in this direction. We modified the end of our discussion as follows to reflect these points:

“*We do not suggest that CapsNets are the only implementation of grouping and segmentation. We only suggest that grouping and segmentation is important and CapsNets are one potential way in which grouping could be solved. Further work is needed to show how the brain implements it. Regardless of the specific implementation, a focus on recurrent grouping and segmentation offers a fresh way of conceptualizing global visual processing and has important implications far beyond crowding, for neuroscience and computational science in general. Harnessing the power and flexibility offered by deep learning approaches beyond classic ffCNNs will be crucial to understand and model these complex processes. We suggest that important new avenues of research will open up when complex deep networks such as CapsNets can be scaled to deal with large complex datasets, and this contribution is an example of this promising approach.”*

**To mention**

**• The experiment has been programmed in an early version of Tensorflow, this will be more difficult to track once the paper is published. Would it be possible to update this code to Tensorflow 2.0, or alternatively, the latest version of Tensorflow 1.x?**

Porting our code to tensorflow 2 would require a *lot* of changes to our code, as we are using many functionalities specific to tensorflow 1.

We checked that our code runs with the latest version of tensorflow 1 (version 1.15).

**Minor points**

**• L53, apart from grouping another major difference, relevant to mention, is that DNNs make different mistakes than humans in classification.**

In this passage, we mention adversarial examples and the texture bias of ffCNNs, which is another way of saying that ffCNNs and humans have different error patterns. Which other work would you suggest citing?

**• The authors might want to point to the developing literature on capsule networks in tumor detection, both highlighting its general use, and its underexposure in CCN.**

Thank you for this suggestion. We added the following sentence to the discussion:

“*Relatedly, CapsNets may also be more resilient to adversarial attacks (57, 58), generalize better to new viewpoints (59), and outperform ffCNNs on difficult visual tasks with scarce training data such as tumor detection (60), also suggesting potentially more human-like generalization than ffCNNs.”*

**• Capsule networks allow you to decode specific information from specific capsules. You can read this as a hack in simulating recurrent processing but also as a feature of higher-tier areas when dealing with lower-tier common input. You could have more shine on the capsule networks here (and this, as far as I can see, really adds to the current corpus of D(R)NNs.**

Thank you. We added the following sentence to page 6:

“*Because each feature is represented in its own capsule, we can decode how each feature of a stim-ulus is represented in its capsule to gain important insights about the underlying computations. This is an advantage compared to ffCNNs, in which it is unclear which neurons represent which feature.”*

**REVIEWER 2:**

**I found this paper very interesting and, overall, I don't think it requires much additional work/revision. From my viewpoint, there is only one major concern, and it is overlap with prior publications from the same group. For example, in a previous publication in the same journal last year (Doerig et al 2019), the authors make similar points, in particular the fact that feedforward architectures like cnn do not explain uncrowding. The manuscript under revision does add to that prior publication, but it is more of an incremental addition than an entirely new concept. As far as I can see, the main contribution is to take an established neural network architecture like capsule nets, and let the capsules do the job that was formerly carried out by LAMINART. It is not quite that simple, and of course capsule nets is an attractive integrated approach rather than a hybrid one, I get that, but if you look at the bigger picture that's more or less correct. I don't think there is a general problem with this approach i.e. that of publishing many papers all inching one step at a time towards a final goal. I personally don't subscribe to this approach, but I understand that it is common in the field and so the authors should not be penalized for conforming to it like many other groups. In the end, it is for editors to judge whether the incremental knowledge is sufficient to justify another publication in PLOS CB or not. As far as I am concerned in my role as reviewer, when I evaluate this paper in and of itself (without comparing it to others by the same group), I think the paper is good and interesting.**

**There are potentially several specific aspects that one could pick upon and demand endless variants of the training	protocol, inclusion of other architectures, and so on. But I don't think that is necessarily productive, because the authors have already done a good job of exploring different aspects and variants of this topic. One obvious issue, which the authors identify as being unresolved, is the separation of crowding stimuli from the vernier target during training. It is clear why the authors used this approach (i.e. to steer the network towards grouping the crowding array as being a separate element than the vernier offset), and it seems sensible. It would be nice if the network could figure that out for itself, but I guess that will be for another paper.**

Thank you for your comments. We added the following new passages to our discussion to stress the novelty of this contribution.

“*The approach we took in this contribution goes beyond the standard use of deep learning in neuro-science, in which large ffCNN are fitted on large datasets to model behaviour. To our knowledge, there is little research on the specific kinds of computations that ffCNNs cannot do and, importantly, why they cannot do it. Here, we have shown that a very specific kind of recurrent computations is needed to explain global processing: recurrent grouping and segmentation. Furthermore, experiment 2 goes beyond the usual static image recognition measures by taking time-extended computations into account. There are currently very few attempts to explain the dynamics of human neural computations using deep learning (with the notable exception of (10)). This may be because most researchers use ffCNNs, which are ill-suited to address such dynamic computations. Contrasting different non-standard deep learning architectures to ask specific neuroscientific questions about well-defined tasks can lead to more precise ways of using deep networks in neuroscience. We think such approaches will be important in the future. We reply to your concerns in detail at the beginning of this letter, which we copy here for convenience.“*

And

“*Regardless of the specific implementation, a focus on recurrent grouping and segmentation offers a fresh way of conceptualizing global visual processing and has important implications far beyond crowding, for neuroscience and computational science in general. Harnessing the power and flexibility offered by deep learning approaches beyond classic ffCNNs will be crucial to understand and model these complex processes. We suggest that important new avenues of research will open up when complex deep networks such as CapsNets can be scaled to deal with large complex datasets, and this contribution is an example of this promising approach.”*

We reply to your concerns in detail at the beginning of this letter, which we copy here for convenience.

“*Reviewer 2 expressed some concerns that our results may potentially not be sufficiently novel with respect to a previous publication. However, there is very little overlap between the two except that psychophysical data come from similar experiments. Whereas the previous contribution was mainly about which models can explain crowding, this manuscript is about global processing and how shortcomings of standard ffCNNs in this domain can be overcome. Since global processing is one of the major hurdles facing current ffCNNs, this manuscript makes an important contribution to the fields of vision science, neuroscience and deep learning.*

*Neuroscience is being deeply impacted by artificial neural networks. However, comparisons between these networks and human processing are usually quite unspecific. Typically, large ffCNN models are compared by simply fitting large datasets. To our knowledge, there is little research on specific kinds of computations that ffCNNs cannot do, and, importantly, why. Here, we show that recurrent computations specifically implementing grouping and segmentation are needed to explain our data. Furthermore, we show that the computational dynamics are well modelled too. For this, we created a psychophysical experiment, which we and reviewer 1 think is interesting in itself because it goes beyond the usual static image recognition measures by taking time-extended computations into account. Strikingly, we found that increasing the time allowed for recurrent computations leads to a better segmentation of the flankers from the target in both humans and CapsNets (but not in ffCNNs and recurrent CNNs). This occurs through a dynamic process during which groups of neurons determine how to segment a stimulus in order to maximize agreement between capsules. Our results pave a new way for challenging neural networks by explicitly looking into the dynamics of processing. We think that paradigms such as ours may become important testbeds for neural network models in general.*

*As reviewer 1 noted, there are very few attempts to explain the dynamics of human neural computations using deep learning (with the notable exception of Kietzmann et al. 2019). This may be because most researchers use ffCNNs, which are ill-suited to address such dynamic computations. Thus, recurrent networks are needed. However, as we mention in our discussion, recurrent networks are still rarely used in neuroscience, and most recurrent networks that are used are simple recurrent extensions of ffCNNS. In fact, this is the first time that CapsNets are used in a neuroscience context (to our knowledge). Therefore, CapsNets are not a well-established model in neuroscience, contrary to what reviewer 2 claimed. We had to develop a large code database to implement CapsNets and test them on psychophysical stimuli, decode vernier offsets, apply the right loss functions, investigate dynamic changes in capsule representations over time and to compare CapsNets with other network architectures.*

*The focus of this contribution is very different from our previous publication (Doerig et al., 2019). In our previous publication, we were interested in crowding specifically. Here, we were interested in general models of global processing, and simply use (un)crowding as a well-controlled probe because this is our experimental expertise. Our results show how CapsNets can flexibly learn to group and segment different parts of the stimulus and why this naturally leads to human-like global processing. Rather than a model geared at explaining this specific psychophysical result, CapsNets offer a general framework to understand human global processing in a wide variety of tasks and modalities (contrary to the LAMINART and its hand-tuned neural connections directly aimed at solving visual uncrowding). Indeed, CapsNets can be applied to any task in principle. This property makes CapsNets general enough to be considered as an alternative framework to the classic conception of the visual system embodied by ffCNNs. Our strong results show that such a framework is a qualitatively better model of human global processing than models based on the classic pooling conception to vision. Future work is needed to tell to what extent CapsNets match human processing in different tasks, opening up exciting new research directions.*

*In summary, our main aim was not only to show that CapsNets can reproduce the (un)crowding results, but to understand how they do it and which kind of recurrent processing is needed in order to do so. This offers a fresh way of conceptualizing global visual processing and has important implications far beyond crowding, for neuroscience and computational science. We think that, when complex deep networks such as CapsNets scale, important new avenues of research will open up, and this is an early example of this promising approach. We added a new paragraph to the discussion explicating some of these points.”*
